# Nonsuicidal self-injury among Chinese university students during the post-COVID-19 era: analysis of sex differences and the impact of gender role conflict

**DOI:** 10.3389/fpsyg.2024.1362762

**Published:** 2024-06-24

**Authors:** Moye Xin, Julia Petrovic, Chengxi Yang, Lijin Zhang, Xueyan Yang

**Affiliations:** ^1^School of Psychology, Shaanxi Normal University, Xi'an, China; ^2^Human Development, McGill University, Montreal, QC, Canada; ^3^School of Liberal Arts, Yulin University, Yulin, Shaanxi, China; ^4^Institute for Population and Development Studies, Xi’an Jiaotong University, Xi’an, Shaanxi Province, China

**Keywords:** non-suicidal self-injury, university students, gender role conflict, sex differences, post-COVID-19 era

## Abstract

**Background:**

Global centers of epidemic prevention and control have entered a new stage of normalization, namely, the “post-COVID-19 era.” During the post-COVID-19 era, which is characterized by the time period following that with the most serious medical consequences, the psychosocial consequences of the COVID-19 pandemic began to receive worldwide attention, especially the degree of psychological distress it caused.

**Aim:**

This study explored the differential impact of gender role conflict on Chinese university students’ engagement in nonsuicidal self-injury (NSSI) as a function of biological sex following the global COVID-19 pandemic.

**Methods:**

Participants were 1,600 university students in northwestern China (*M*_age_ = 21.3 years; 50.8% women) who completed online measures of demographic variables (including biological sex, gender role conflict, and NSSI engagement).

**Results:**

Women reported significantly more gender role conflicts than men did, while engagement in NSSI was significantly more prevalent among men than women. A total of 262 men reported engaging in at least one NSSI behavior, resulting in a prevalence rate of 33.25%. In comparison, a total of 106 individuals reported engaging in at least one NSSI behavior, resulting in a prevalence rate of 13.05% among women. Gender role conflict was found to significantly predict university students’ NSSI engagement, regardless of biological sex.

**Conclusion:**

This is the first empirical study to identify sex differences in both gender role conflict and engagement in NSSI among university students in Northwestern China during the post-COVID-19 era. In addition, the present study is the first to demonstrate how gender role conflict predicts engagement in NSSI across sexes. These findings will inform the literature on gender role conflict and NSSI, particularly the close relationship between gender role conflict and engagement in NSSI among Chinese university students, and they emphasize the need for continued efforts to explore NSSI cross-culturally.

## 1 Introduction

### 1.1 NSSI in the post-COVID-19 era

The outbreak of the COVID-19 pandemic in the beginning of 2020 had severe impacts on society and the global economy, in addition to disrupting people’s daily routines and patterns. At present, global centers of epidemic prevention and control have entered a new stage of normalization, namely, the “post-COVID-19 era” (Sun et al., 2022). During the post-COVID-19 era, which is characterized by the time period following that with the most serious medical consequences, the psychosocial consequences of the COVID-19 pandemic began to receive worldwide attention, especially the degree of psychological distress it caused (Bates et al., 2021). For instance, Chinese citizens who frequently experienced high levels of psychological distress during the pandemic now report significantly greater mental health deterioration than people who did not (Wang et al., 2022). Furthermore, university students appeared to be disproportionately negatively impacted by social isolation and day-to-day uncertainty resulting from the pandemic; in fact, they reported lower psychological resilience during and after the pandemic, making them more prone to negative emotions (e.g., fear, anger, anxiety, restlessness, etc.) than adults in other occupations (e.g., workers, teachers, doctors, etc.) and age groups (e.g., seniors, children, etc.). In sum, these findings suggest that university students may continue to experience varying degrees of psychological distress in the post-COVID-19 era, and their overall mental health and coping abilities may also vary as a result. Some students may engage in healthy coping behaviors to manage their stress, which can promote mental health (Stallman et al., 2020). In contrast, other university students may adopt unhealthy coping behaviors, such as nonsuicidal self-injury (Stallman, 2020), which might exacerbate their levels of perceived stress and mental health difficulties (Christoforou et al., 2021).

NSSI (Non-suicidal self-injury) refers to deliberate self-inflicted damage to one’s own body tissue by methods such as cutting, scratching, and self-hitting that leads to tissue damage, without conscious suicidal intent and for reasons not socially sanctioned. NSSI is commonly used among youth as a method of dealing with painful emotions and thoughts (Xin et al., 2022) and can have varying degrees of negative consequences. It is a prevalent health concern among university students, given that they are twice as likely to engage in NSSI than their nonuniversity attending peers are (Swannell et al., 2014). Among Western individuals, approximately 20–30% of university students reported a history of NSSI (Wester and King, 2018), and 10–15% of university students engaged in NSSI for the first time during their university years (Kiekens et al., 2019).

Before the pandemic, the prevalence of NSSI among Chinese youth was already higher than that among comparable Western individuals, with prevalence rates fluctuating within a relatively large range (between 31.8 and 36.3%; Xin et al., 2020). However, whether the psychological consequences of the COVID-19 pandemic have impacted the prevalence of NSSI among Chinese university students and whether there are sex differences in engagement in NSSI during the post-COVID-19 era remain unclear. The impact of gender role conflict on mental health outcomes, such as non-suicidal self-injury (NSSI), has been an emerging area of concern for mental health professionals. Prior research indicates a complex relationship between gender roles and adverse psychological effects. However, the nuances of this dynamic within various cultural backgrounds, especially during unprecedented times such as the post-COVID-19 era, are not thoroughly understood. The present study aims to address this gap by investigating the potential sex differences in the relationship between gender role conflict and engagement in NSSI among university students in Northwestern China. By applying a cross-cultural lens, we attempt to understand how these variables interact within a specific regional context compared to existing literature from varied cultural backgrounds, offering a comparative perspective that could reveal how cultural factors influence the manifestation and perception of gender roles and their impact on mental health. The current study seeks to illuminate the intersection between gender role conflict and NSSI, exploring potential sex differences among university students in Northwestern China. By adopting a cross-cultural perspective, our research aspires to unravel the ways in which cultural factors shape gender roles and their subsequent impact on mental health, contrasting these phenomena with findings from other cultural settings. This endeavor aims to produce insights that will underpin the creation of culturally sensitive and gender-responsive mental health interventions tailored to the unique needs of this population, hence offering a clear purpose to the academic community and practical benefits to public health practitioners.

### 1.2 Gender role conflict and NSSI

Gender role conflict is a construct based on the idea of gender roles; it refers to the personality traits, psychological states, emotional attitudes, and behavioral patterns that individuals are expected to exhibit in accordance with their biological sex, which result from processes of socialization within a certain cultural and ideological context (Xu, 2016). Gender role conflict, then, is defined as a psychological state in which socialized gender roles have negative consequences for oneself and others (O’Neil, 2008). Gender role conflict occurs when rigid, sexist, or restrictive gender roles result in devaluations, restrictions, or violations against the self and others that limit human potential and freedom. Among men, gender role conflict has historically been related to the following five areas: physical deficiencies, emotional exposure, female subordination, mental illness, and performance failure (Eisler and Skidmore, 1987). Among women, gender role conflict has been linked to the following five areas: rational relationships, physical unattractiveness, victimization, decisive behavior, and peripheral support relationships (Gillespie and Eisler, 1992).

The experience of gender role conflict can result in a variety of negative consequences for university students and their families. First, previous research has demonstrated a robust link between gender role conflict and mental health difficulties, as well as between gender role conflict and barriers to help-seeking, among university students (e.g., Yang et al., 2013; Lu et al., 2015). Previous research with university students has demonstrated that men with high levels of gender role conflict are likely to experience negative emotions such as stress and depression (Jacobson et al., 2011). Similarly, Lu et al. (2015) found that gender role conflict is closely related to mental ill-being among men attending university. In addition, studies have revealed that individuals with high levels of gender role conflict, compared to those with low levels of gender role conflict, are more likely to adopt a negative attitude toward psychological counseling and related therapies in the face of mental health difficulties, avoid help-seeking, and refuse treatment (Yang et al., 2013). Second, gender role conflict may lead to social exclusion, causing university students with such conflict to isolate themselves from social groups, resulting in challenges in interpersonal relationships, daily life, learning, and work (Wu et al., 2016). Hayes and Mahalik (2000) found that men with high levels of gender role conflict were afraid to express their feelings and communicate with others, and that these men experienced significant obstacles in establishing and maintaining interpersonal relationships. Third, gender role conflict is a risk factor for self-inflicted physical bodily harm (i.e., NSSI, suicide), which can be detrimental to the physical and mental health of those experiencing gender role conflict and to their families (Yang et al., 2015).

Significant relationships between NSSI and constructs related to gender role conflict have been documented in previous studies. For example, Hawton et al. (2002) conducted a cross-sectional study with 6,020 high school students in the United Kingdom and reported that boys and girls who reported sexual orientation confusion also reported a greater prevalence of NSSI than did their peers who did not report such confusion. Similarly, Whitlock et al. (2006) conducted research with 8,300 university students in the U.S. and reported that self-reported bisexuality and/or confusion about sexual orientation were significantly positively associated with repetitive NSSI. Finally, studies have revealed that gender role identity confusion has a significant impact on the prevalence of NSSI among university students (Stanley et al., 2001). In sum, these studies suggest that there might be an important link between gender role conflict and the prevalence of NSSI among university students.

### 1.3 Research gaps

**Gap 1:** With regard to the measurement of gender role conflict, due to the imperfect cross-cultural applicability of existing scales, there is a lack of measurement scales that are suitable for China’s sociocultural context. In addition, measurement scales of gender role conflict have been largely centered around the experiences of men in China (Kang et al., 2016), resulting in a lack of measurement scales that are applicable to women, largely restricting the comparative study of gender role conflict as a function of biological sex. Finally, previous Chinese studies have paid more attention to the causes of gender role conflict (Xie et al., 2023; Zheng, 2023) and less attention to the negative consequences of gender role conflict across different developmental periods.

**Gap 2:** In the NSSI literature, there is a lack of studies that assess the prevalence of NSSI among Chinese university students using measurement scales that take into account NSSI-related behavioral trends in China. The vast majority of related research on the measurement of university students’ NSSI behaviors has concentrated on Western samples (Whitlock, 2011; Burke et al., 2017; Simone and Hamza, 2021; Holden et al., 2022), and Chinese scholars’ NSSI scale development has been largely based on the translation and slight revision of Western scales (Qiu and Hou, 2020). This has led to significant deficiencies in NSSI research based on the Chinese sociocultural context, thereby rendering comparative research on self-injury domestically and internationally challenging.

**Gap 3:** There is a consensus that the prevalence of NSSI among young people in China has increased significantly since the onset of COVID-19 (Li and Wang, 2021). However, there are mixed views regarding whether there are sex differences in the prevalence of NSSI in the post-COVID-19 era. Prior to the COVID-19 pandemic, many studies noted significant sex differences in the prevalence of NSSI among university students (Claes et al., 2007; Nixon et al., 2008). Several international researchers have reported that the prevalence of NSSI was greater among women than among men (Lundh et al., 2011). For example, Bresin and Schoenleber (2015) reviewed many relevant studies in the last century and found that the prevalence of NSSI among women was 1.5 to 3 times greater than that among men. However, several Chinese researchers have found that compared with women, men are more inclined to engage in NSSI. For instance, studies by Yang et al. (2015) and Zhang and Wang (2015) revealed that the incidence of NSSI was significantly greater among men than among women. During the post-COVID-19 era, many researchers refuted the possibility of sex differences in the prevalence of NSSI among university students. According to Wei et al. (2021), biological sex does not play a substantive role in determining the prevalence of NSSI among university students. Relatedly, through an anonymous survey of 371 university students, Hou (2022) found that there was no significant difference in the prevalence of NSSI as a function of biological sex.

In summary, engagement in NSSI among university students became a global public health concern before the onset of the COVID-19 pandemic. Nevertheless, the pandemic has exacerbated many of the mental health difficulties that were, in part, underlying engagement in NSSI (Nurseskasatmata et al., 2020; Zhang et al., 2020). Thus, there is an urgent need to explore potential risk factors for engagement in NSSI among Chinese university students to better inform prevention and intervention efforts. Moreover, gender role conflict has been suggested to be a potential risk factor for university students’ engagement in NSSI (Yang et al., 2015); however, additional research is needed to clarify the relationship between these two variables in the current postpandemic context. Therefore, the present study assessed two main research objectives. The first objective was to assess sex differences in (a) gender role conflict and (b) NSSI prevalence among Chinese university students. The second objective was to determine the potential impact of gender role conflict on university students’ engagement in NSSI as a function of biological sex to determine whether gender role conflict is a risk factor for self-injury.

## 2 Materials and methods

### 2.1 Sample and control of responses

Participants were drawn from a stratified, random sample of Chinese university students, with measures in place to ensure a representative demographic spread and to minimize sampling bias. Multiple submissions from the same participant were controlled using unique identifiers without compromising participant anonymity. As a measure to prevent response duplication and to ensure data integrity, each submission was electronically tagged with a non-identifiable participant code that allowed for tracking of multiple submissions from the same source. Participants were a sample of 1,600 students (788 men, 812 women; *M*_age_ = 21.3 years; age range: 17–24 years) recruited from four universities in Northwestern China from four university disciplines: science, engineering, medicine, and liberal arts. Specifically, students were recruited from 11 departments, including the school of science (9.5% of participants), school of mechanical engineering (10.5%), school of electrical engineering (8.2%), school of energy and power engineering (7%), school of electronic and information engineering (9.4%), school of life sciences and technology (10.1%), school of aerospace and aeronautics (7.3%), school of medicine (6.8%), school of economics and finance (6.4%), school of public policy and management (11.8%), and school of humanities and social sciences (13%). Proportional stratified sampling was used to recruit men and women across levels of study (i.e., freshmen, sophomores, and juniors) from the four participating universities. Of the total sample, 32.1% were freshmen, 33.3% were sophomores, and 34.4% were juniors. For participants under the age of 18, parents or legal guardians gave permission for students’ participation by signing consent forms prior to the survey. The survey was completely anonymous and was conducted online. A total of 1,700 questionnaires were distributed, and 1,600 valid responses were collected, resulting in a completion rate of 94.1%. All procedures were approved by the Institutional Review Board of the school of Psychology, Shaanxi Normal University.

### 2.2 Measures

**Gender role conflict**: In this investigation, the original Masculine Gender Role Stress Scale (MGRS; Eisler and Skidmore, 1987) and the Feminine Gender Role Stress Scale (FGRS; Gillespie and Eisler, 1992) were revised for assessing gender role conflict among Chinese university students. Keeping with the original scales’ structure and items, the adapted MGRS includes 40 items across five domains: physical deficiency (9 items), emotional exposure (7 items), female subordination (9 items), mental retardation (7 items), and performance failure (8 items). Similarly, the adapted FGRS encompasses 35 items spanning five domains: fear of rational relationships (5 items), fear of physical unattractiveness (13 items), fear of victimization (6 items), fear of decisive behavior (7 items), and fear of lack of supportive relationships (4 items). Both scales employ a 5-point Likert response format ranging from 1 = Totally disagree to 5 = Fully agree. In this study, Cronbach’s alpha indicated high internal consistency for the MGRS (α = 0.89) and FGRS (α = 0.91).

**NSSI engagement**: Engagement in NSSI was measured using a culturally adapted Chinese translation of the NSSI-AT (Whitlock et al., 2014). Participants responded to the question, “Have you ever engaged in the following behaviors, without the intent of suiciding?,” with response options of 0 = no and 1 = yes. To capture culturally specific NSSI behaviors, four additional items supportive of Chinese youth experiences were incorporated: (1) Ingesting indigestible objects; (2) overmedicating; (3) self-burning with cigarettes; and (4) creating ‘blood tattoos’ with corrosive substances or engraving. This resulted in a comprehensively modified 16-item assessment (see revised Table 1). The internal consistency for this adapted scale within the current study was solid (α = 0.82).

**Table 1:** Modified NSSI-AT to reflect the Chinese context.

	NSSI behaviors
1	Severely scratching the skin with fingernails or other objects (e.g., glass, thumbtacks, etc.), causing bleeding and leaving scars
2	Cutting the wrist, arm, leg, or other part of the body
3	Engraving characters or non-professional tattoos, or dropping corrosive substances on the skin to make a “blood tattoo”
4	Ingesting corrosive substances or swallowing sharp objects
5	Biting yourself and bleeding, resulting in scars on the skin
6	Trying to break your own bone, but failing
7	Successfully breaking your bone
8	Swallowing items that cannot be digested such as plastic, stone, etc.
9	Burning yourself with cigarette butts
10	Stabbing or lashing yourself until bruised or bleeding
11	Bashing or stabbing an old wound
12	Deliberately preventing wound healing
13	Engaging in boxing or other violent activities and deliberately injuring yourself
14	Pulling your hair, eyelashes, or eyebrows consciously and violently
15	Taking or swallowing too much medicine (beyond medical recommendations)
16	Other

**Control variables**: In order to further enrich the validation model, this study selected a large number of demographic variables as control variables. The control variables involved and their measurement methods are shown in Table 2.

**Table 2:** Control variables and their measurements.

Variables	Measurements	Contents (corresponding to questionnaire options)
Age	0 = 18 & under	
	1 = above 18	
Single child or not	0 = single child	
	1 = not single	
Level of study	0 = freshman	
	1 = sophomore	
	2 = junior	
Major	0 = Science & engineering	Science, mechanical, electrical, energy & power engineering, telecommunications, life science, aerospace
	1 = Medicine	Medicine
	2 = Liberal arts & social science	Economics, finance, public management, humanities and social sciences
Place of origin	0 = Western	Chongqing, Sichuan, Guizhou, Yunnan, Shaanxi, Gansu, Qinghai, Inner Mongolia, Xinjiang, Tibet, Ningxia, Guangxi
	1 = Central	Shanxi, Anhui, Jiangxi, Henan, Hubei, Hunan
	2 = Eastern	Beijing, Tianjin, Shanghai, Hebei, Zhejiang, Fujian, Shandong, Guangdong, Hainan, Jilin, Liaoning, Heilongjiang, Jiangsu
Fathers’ educational level	0 = Secondary school and below	No schooling, primary school, junior high school, senior high school
	1 = University and above	Junior university, undergraduate, Graduate
Mothers’ educational level	0 = Secondary school and below	No schooling, primary school, junior high school, senior high school
	1 = University and above	Junior university, undergraduate, Graduate

**Biological sex**: Biological sex was assessed binarily using a single item that asked, “What is your biological sex?.” The response options included “Man” or “Woman.”

### 2.3 Data analysis strategy

Objective 1: Descriptive statistics (e.g., mean, standard deviation, minimum, maximum) were used to assess the current state of gender role conflict among men and women attending universities in China. Independent sample t tests were used to assess differences between men’s and women’s degrees of gender role conflict, while chi-square tests were used to compare NSSI incidence between men and women.

Objective 2: To assess the impact of overall gender role conflict on university students’ engagement in NSSI (i.e., the presence or absence of a history of NSSI) as a function of biological sex, separate binary logistic regression analyses were conducted for men and women. For men, overall gender role conflict was first entered as the sole independent variable, and NSSI was entered as the binary dependent variable (Model 1). In a subsequent step, the analysis was repeated with the inclusion of demographic control variables (i.e., age, only child status, level of study, major, place of origin, father’s educational level, and mother’s educational level) to construct a hierarchical regression model (Model 2). This two-step analytic approach was then repeated for women (Models 3 and 4).

## 3 Results

### 3.1 Descriptive analysis results

Table 3 presents descriptive statistics related to men’s gender role conflict. The average value of men’s gender role conflict was 2.31, suggesting that men tended to report relatively low levels of gender role conflict (given the possible score range of 1 to 5). According to the descriptive statistics for the five dimensions of gender role conflict, men’s reported degree of gender role conflict appeared to be roughly similar across the five dimensions, with mean scores ranging from 2.19 to 2.38.

**Table 3:** Descriptive analysis of gender role conflict among men attending university.

	Sample size	Min	Max	Mean	Standard deviation
Physical deficiency (9 items)	788	1.00	5.00	2.36	0.71
Emotional invisibility (7 items)	788	1.00	4.29	2.38	0.68
Female subordination (9 items)	788	1.00	4.33	2.19	0.62
Mental illness (7 items)	788	1.00	4.29	2.27	0.68
Performance failure (8 items)	788	1.00	4.88	2.34	0.81
**MGRS (Overall)**	**788**	**1.00**	**3.73**	**2.31**	**0.57**

Table 4 provides descriptive statistics related to women’s gender role conflict. The average score for gender role conflict among women was 3.18, suggesting that women tended to report a slightly greater degree of gender role conflict than men did. The greatest degree of conflict was reported in the areas of fear of rational relationships, fear of victimization, and fear of peripheral support relationships. A relatively low degree of gender role conflict was reported in the areas of fear of physical unattractiveness and fear of decisive behavior.

**Table 4:** Descriptive analysis of gender role conflict among women attending university.

	Sample size	Min	Max	Mean value	Standard deviation
Fear of rational relationships (5 items)	812	1.00	5.00	3.89	0.67
Fear of physical unattractiveness (13 items)	812	1.00	4.88	2.78	0.60
Fear of victimization (6 items)	812	1.00	4.89	3.20	0.69
Fear of decisive behavior (7 items)	812	1.00	5.00	2.97	0.60
Fear of peripheral support relationships (4 items)	812	1.00	5.00	3.06	0.82
**FGRS (Overall)**	**812**	**1.66**	**4.51**	**3.18**	**0.48**

Table 5 displays the results of an independent sample t test comparing the mean levels of gender role conflict between men and women. It can be inferred that the gender role conflict reported by women was significantly greater than that reported by men (*p* < 0.001).

**Table 5:** Comparative analysis of gender role conflict among university students as a function of biological sex.

	Men (*N* = 788)		Women (*N* = 812)		*t*-value
	Mean	Standard deviation	Mean	Standard deviation	
Gender role conflict	2.31	0.57	3.18	0.48	*t* = −21.63*

***p < 0.001, **p < 0.01, *p < 0.05, *^+^p* < 0.1.

Table 6 presents the results of a chi-square test assessing potential differences in the prevalence of NSSI among university students as a function of sex. A total of 368 university students reported engaging in at least one NSSI, resulting in an overall prevalence of NSSI of 23.00%. A total of 262 men reported engaging in at least one NSSI behavior, resulting in a prevalence rate of 33.25%. In contrast, a total of 106 women reported engaging in at least one NSSI, resulting in a 13.05% prevalence among women. A chi-square test revealed that this sex difference in engagement in NSSI was statistically significant at the 90% confidence level, suggesting that the prevalence of NSSI among men was comparatively greater than that among women.

**Table 6:** Comparative analysis of NSSI prevalence among university students as a function of biological sex.

	Men (N = 788)		Women (N = 812)		Total (N = 1,600)	
	Frequency	Percentage (%)	Frequency	Percentage (%)	Frequency	Percentage (%)
0 = No NSSI	526	66.75	706	86.95	1,232	77.00
1 = NSSI	262	33.25	106	13.05	368	23.00
χ^2^	4.23^+^					

***p < 0.001, **p < 0.01, *p < 0.05, ^+^*p* < 0.1.

### 3.2 Regression analysis results

Table 7 displays the binary logistic regression results, which assessed the role of overall gender role conflict in predicting the presence or absence of a history of NSSI engagement. In Model 1, gender role conflict emerged as a significant positive predictor of engagement in NSSI among men (*β* = 1.63, *p* < 0.001). Demographic control variables were then added to Model 2; these included age, only child status, level of study, major, place of origin, father’s educational level, and mother’s educational level. According to Model 2, gender role conflict remained a significant positive predictor of engagement in NSSI among men (*β* = 2.34, *p* < 0.001). Of all the control variables included in the model, only the level of study emerged as a significant positive predictor of engagement in NSSI among men (*p* < 0.10). Compared to freshmen, sophomores and juniors were more likely to have engaged in NSSI.

**Table 7:** Regression analysis of the impact of overall gender role conflict on students’ NSSI.

Dependent variable: NSSI or no-NSSI (Benchmark: no)	Model 1 (Men)	Model 2 (Men)	Model 3 (Women)	Model 4 (Women)
Independent variable				
Gender role conflict (overall)	1.63***	2.34***	0.82*	1.44**
Control variables				
Age (Benchmark: 18 and below)				
18 and above		−0.19		1.79**
Single child or not (Benchmark: not)				
Single child		−0.22		−0.16
Grades (Benchmark: Freshmen)				
Sophomore		0.64^+^		0.19
Junior		0.87^+^		−0.27
Majors (Benchmark: Science & engineering)				
Medicine		0.04		−0.04
Liberal arts & social science		−0.19		−0.32
Place of origin (Benchmark: Western)				
Central		−0.13		0.19
Eastern		−0.32		0.12
Father’s educational level (Benchmark: Secondary school and below)				
University and above		−0.17		0.14
Mother’s educational level (Benchmark: Secondary school and below)				
University and above		0.29		−0.06
−2 Log Likelihood	513.57***	437.43***	443.61***	383.16***
Cox & Snell R^2^	0.07	0.09	0.05	0.13
Nagelkerke R^2^	0.07	0.12	0.09	0.13

***p < 0.001, **p < 0.01, *p < 0.05, ^+^ *p* < 0.10.

From Model 1 to Model 2, the value of the −2 log likelihood decreased from 513.57 to 437.43, indicating that the degree of fit of the regression model improved. Moreover, as the Cox and Snell R^2^ increased from 0.07 to 0.09, the Nagelkerke R^2^ increased from 0.07 to 0.12, indicating that the degree to which the independent variables explained the variation in the dependent variables improved, and the explanatory power of the regression model was strengthened.

In Model 3, overall gender role conflict emerged as a significant positive predictor of engagement in NSSI among women (*β* = 0.82, *p* < 0.05). Demographic control variables (i.e., age, only child status, level of study, major, place of origin, father’s education level, and mother’s education level) were then added to Model 4. In Model 4, gender role conflict continued to emerge as a significant positive predictor of engagement in NSSI among women (*β* = 1.44, *p* < 0.01). None of the demographic control variables emerged as significant predictors of NSSI engagement.

From Model 3 to Model 4, the value of the −2 log likelihood decreased from 443.61 to 383.16, indicating that the degree of fit of the regression model improved. Moreover, as the Cox and Snell R^2^ increased from 0.05 to 0.13, the Nagelkerke R^2^ increased from 0.09 to 0.13, indicating that the degree to which the independent variables explained the variation in the dependent variables improved, and the explanatory power of the regression model was strengthened.

## 4 Discussion

### 4.1 Descriptive analysis discussion

The primary aim of our investigation was to examine gender differences in the experience of gender role conflict and the prevalence of nonsuicidal self-injury (NSSI) among university students in China. The study found that female students reported significantly higher levels of gender role conflict compared to their male counterparts. Our analysis indicates that the intersection of rapid economic and social transformation in China, especially in light of the recent COVID-19 pandemic, may be influencing these reported differences (Lu et al., 2015). Furthermore, despite emerging trends toward greater gender equality, the remnants of a deep-seated patriarchal culture may still exert substantial influence on gender roles (Yang et al., 2011; Liu, 2018). These observations are in line with Xu (2022), who noted that the cultural context, particularly in a male-dominated society such as China’s, can significantly impact an individual’s experience of gender role conflict, with young women potentially facing more acute challenges in this regard.

The present findings also revealed that the prevalence of NSSI among university students was 23%; that is, 23 out of every 100 university students engaged in NSSI at least once in their lifetime. Within Western contexts, the prevalence of NSSI among university students has been shown to fluctuate between 20 and 30% (Bjärehed and Lundh, 2008; Hilt et al., 2008). According to previous studies of Chinese university students, the prevalence of NSSI fluctuates between 31.8 and 36.3% (Xin et al., 2020; Zhao et al., 2021; Zhang et al., 2022). Considering the sociocultural differences between previous research and the present study, as well as differences in measurement methods, the prevalence of NSSI among university students involved in this study is still relatively high compared with that in previous national and international studies. Particularly, compared with that of patients before the onset of the COVID-19 pandemic (Yang and Xin, 2018), the prevalence of NSSI appears to have increased, which warrants further exploration into current risk and protective factors.

In the present study, the prevalence of NSSI among men was unexpectedly higher than that among women. This novel finding implies that in the post-COVID-19 era, men may be encountering unique challenges that contribute to their engagement in NSSI behaviors, aligning with recent research suggesting shifting patterns (Li and Wang, 2021). It is important to note, however, that the domain of sex differences in NSSI prevalence is one of ongoing debate and varying conclusions in academic discourse (Fu et al., 2017). As such, while our findings contribute to this complex picture by indicating higher NSSI rates among men, interpretations of these results must remain tentative and be considered within the broader, often contradictory, context of existing research. One potential interpretation could be the differential impact of the pandemic on mental health by gender. It is plausible that the social and economic repercussions of COVID-19 have heightened stressors that disproportionately affect men, such as pressures related to unemployment or underemployment, which have been exacerbated during this period. These pressures may be particularly consequential given the traditional societal expectations of men as providers. Furthermore, there might be a cultural shift where increased awareness and declining stigma around mental health issues encourage more men to report NSSI behaviors (Li et al., 2021). Additionally, it is worth considering whether the modes of coping with stress and distress may have evolved, with some men possibly turning to NSSI as a coping mechanism in the absence of more traditional support systems, which may have been disrupted due to the pandemic.

### 4.2 Regression analysis discussion

The second objective of the present study was to determine the potential impact of gender role conflict on university students’ engagement in NSSI as a function of biological sex. The findings suggest that there is a close relationship between gender role conflict and university students’ engagement in NSSI during the current post-COVID-19 era, regardless of biological sex. As noted earlier, previous research in China has focused more heavily on the causes of gender role conflict rather than its potential negative consequences (Xie et al., 2023; Zheng, 2023). The present findings are a novel contribution to the gender role conflict and NSSI literature and suggest that gender role conflict may be a risk factor for engagement in NSSI among men and women attending universities in China. Overall, these results suggest that gender role conflict may be an important variable for further exploration of mental health and well-being on Chinese university campuses. Indeed, studies have shown that both men and women attending universities have suffered from negative impacts from gender role conflict in recent years (Chen and Wu, 2021). Nevertheless, given that this was the first study to explore the relationship between gender role conflict and NSSI in Northwest China after the pandemic, additional research is needed to build upon our findings and confirm the temporal relationship between these two variables.

Incorporating a feminist perspective, we examine the relationship between levels of study and NSSI engagement among men, suggesting that societal pressures related to gender roles may exacerbate stress during university years (Brown and Gilligan, 1992). To this point, more advanced university men might struggle with the expectation of self-reliance and may engage in NSSI as a coping strategy in the face of academic and professional pressures that intensify with each academic level (Courtenay, 2000). Moreover, an inclusive examination of gender identity and related conflicts offers a more nuanced understanding of NSSI behaviors (Testa et al., 2017). Gender-diversity may encounter unique stressors, including lack of social support and discrimination, which can contribute further to the engagement in play of these factors can lead to an expanded model of gender role conflict, where the impact of societal expectations on NSSI among university students is viewed within a broader, more inclusive scope of gender experiences. Future research should consider longitudinal designs to fully capture NSSI’s temporal relationship with gender role and identity stressors throughout the university trajectory. Additionally, interventions focusing on the reduction of gender role conflict and the enhancement of healthy coping strategies might prove essential in addressing NSSI among university students.

Finally, of all the demographic control variables explored (i.e., age, only child status, level of study, major, place of origin, father’s educational level, and mother’s educational level), only level of study emerged as a significant positive predictor of NSSI engagement, and this was only the case among men. This finding suggested that the further men progress in their university studies, the more likely they are to report a history of NSSI engagement. A possible explanation for this finding is that men attending university may be engaging in NSSI for the first time during their university years, particularly in later years. This argument is consistent with research demonstrating that the onset of NSSI—while most common during adolescence—often experiences a second “peak” that overlaps with the university years (Whitlock et al., 2011; Gandhi et al., 2017). This phenomenon may be driven by the fact that men at different levels of study are facing different academic and professional pressures (Xu et al., 2021). Freshmen are in a transition period between high school and university, which is often initially perceived as a positive and exciting change despite inevitable challenges (Dai et al., 2020). In contrast, the pressures faced by more senior students (i.e., sophomores and juniors), such as completing more complex courses, successfully completing a stage or field work, writing a thesis, searching for a job, or applying to pursue higher education, may result in greater levels of stress relative to freshmen. Unsurprisingly, these students reported varying degrees of burnout during their later university years (Duan et al., 2017). Notably, stressful experiences during the university years have been found to predict engagement in NSSI among students (Hamza et al., 2021). Taken together, these factors may explain why sophomores and juniors are at high risk in terms of their engagement in NSSI (Li et al., 2018).

## 5 Limitations and future directions

The present findings should be interpreted within the context of the study’s limitations. First, the representativeness of the present sample is limited. From the perspective of geographical distribution, this study used a sample of university students from Xi’an, Shaanxi Province. The results reflect the situation of university students residing in Western China’s provinces and cities, but the applicability of these findings to students in Central and Eastern China’s provinces and cities is limited and further research is warranted. Moreover, relevant studies have shown that social psychological variables, such as psychiatric disorders and borderline personality, can indeed play a critical role in influencing NSSI behavior (Eckert et al., 2021; Scamaldo et al., 2021; Valencia and Sinambela, 2021; Kim et al., 2015). However, these factors were not discussed in the present study. Therefore, further research is needed to delve into the potential relationships between these variables, thereby enhancing the academic rigor of the study. In addition, the approach used to analyze university students’ engagement in NSSI was limited. This study focused on NSSI incidence (i.e., whether or not university students reported a history of engaging in NSSI) and its relation to biological sex and gender role conflict but did not explore the latter two variables in relation to NSSI recency, frequency, severity, age of onset, motivation, consequences, or disclosure. Future studies may further explore the relationship between these variables. Finally, relatively few explanatory variables were included in the regression models. The primary aim of this study was to explore the influence of gender role conflict and demographic control variables on university students’ engagement in NSSI in the context of the postpandemic era. However, other relevant variables (e.g., stressful life events, life satisfaction, emotion regulation, social connectedness, social support, physical exercise, etc.) that may be related to university students’ engagement in NSSI were not explored. Additionally, the present study is cross-sectional designed, which precluded the collection and analysis of longitudinal data. Accordingly, our findings are limited to associations observed at a single point in time during the pandemic and do not encompass how these relationships may have evolved from before the onset of the pandemic. Due to this temporal constraint, we were unable to accurately gage the baseline status of our participants’ experiences, conditions, or attitudes prior to the pandemic, nor could we measure the change over time in these domains. The inability to consider pre-pandemic data in our analysis means that we cannot establish causal inferences from any observed differences or trends, nor can we identify the directionality of the relationships observed. We recognize that this represents a significant gap in the narrative that could potentially be filled by a study design that enables a before-and-after comparison, ideally with the same cohort of participants. Retrospective data collection post-pandemic would introduce recall biases and present methodological difficulties, such as selective memory and changes in perception over time, which could potentially distort the pre-pandemic baseline. We wish to highlight this limitation to underscore the need for caution when interpreting our findings. The study’s scope is therefore restricted to the impact of the pandemic at the specific time of data collection, and we caution against extrapolating these findings to infer changes or trends over the entire course of the pandemic. Future research endeavors should aim to incorporate longitudinal methods when feasible, to better understand the dynamics and causal relationships related to the impacts of the pandemic over time. In future research, the authors will strive to address the above shortcomings, obtain more representative Chinese samples, establish richer models for predicting engagement in NSSI among university students, and assess a wider variety of NSSI-related outcomes.

## 6 Conclusion and implications

The present study was the first to explore potential sex differences in the relationship between gender role conflict and engagement in NSSI among university students in Northwestern China during the post-COVID-19 era. The findings revealed that women reported more gender role conflicts than men did, while men reported a greater prevalence of engagement in NSSI than women did. Moreover, gender role conflict was found to significantly predict university students’ engagement in NSSI regardless of biological sex, suggesting that such conflict may be a risk factor for NSSI among Chinese university students. Additionally, among men, the level of study also emerged as a significant predictor of engagement in NSSI and overall gender role conflict. These findings extend the previous literature on gender role conflict and NSSI and emphasize the need for continued efforts to explore engagement in NSSI and its risk factors cross-culturally. Furthermore, these results highlight the potential importance of addressing gender role conflict in efforts to prevent engagement in NSSI, particularly throughout the university years. Finally, our findings also suggest that university mental health or counseling centers are crucial resources for students experiencing gender role conflict, NSSI, or any other health risks in the context of the current post-COVID-19 era.

## Data availability statement

The raw data supporting the conclusions of this article will be made available by the authors, without undue reservation.

## Ethics statement

The studies involving humans were approved by School of Psychology ethic committee, Shaanxi Normal University. The studies were conducted in accordance with the local legislation and institutional requirements. Written informed consent for participation in this study was provided by the participants’ legal guardians/next of kin. Written informed consent was obtained from the individual(s), and minor(s)' legal guardian/next of kin, for the publication of any potentially identifiable images or data included in this article.

## Author contributions

MX: Conceptualization, Formal analysis, Funding acquisition, Investigation, Writing – original draft, Writing – review & editing. JP: Writing – review & editing. CY: Formal analysis, Investigation, Writing – review & editing. LZ: Supervision, Writing – review & editing. XY: Project administration, Visualization, Writing – review & editing.
